# Nitrate Sensing and Metabolism Inhibit Biofilm Formation in the Opportunistic Pathogen *Burkholderia pseudomallei* by Reducing the Intracellular Concentration of c-di-GMP

**DOI:** 10.3389/fmicb.2017.01353

**Published:** 2017-07-25

**Authors:** Mihnea R. Mangalea, Brooke A. Plumley, Bradley R. Borlee

**Affiliations:** Department of Microbiology, Immunology, and Pathology, Colorado State University, Fort Collins CO, United States

**Keywords:** *Burkholderia pseudomallei*, biofilm, sodium nitrate, c-di-GMP, melioidosis

## Abstract

The opportunistic pathogen *Burkholderia pseudomallei* is a saprophytic bacterium and the causative agent of melioidosis, an emerging infectious disease associated with high morbidity and mortality. Although melioidosis is most prevalent during the rainy season in endemic areas, domestic gardens and farms can also serve as a reservoir for *B. pseudomallei* during the dry season, in part due to irrigation and fertilizer use. In the environment, *B. pseudomallei* forms biofilms and persists in soil near plant root zones. Biofilms are dynamic bacterial communities whose formation is regulated by extracellular cues and corresponding changes in the nearly universal secondary messenger cyclic dimeric GMP. Recent studies suggest *B. pseudomallei* loads are increased by irrigation and the addition of nitrate-rich fertilizers, whereby such nutrient imbalances may be linked to the transmission epidemiology of this important pathogen. We hypothesized that exogenous nitrate inhibits *B. pseudomallei* biofilms by reducing the intracellular concentration of c-di-GMP. Bioinformatics analyses revealed *B. pseudomallei* 1026b has the coding capacity for nitrate sensing, metabolism, and transport distributed on both chromosomes. Using a sequence-defined library of *B. pseudomallei* 1026b transposon insertion mutants, we characterized the role of denitrification genes in biofilm formation in response to nitrate. Our results indicate that the denitrification pathway is implicated in *B. pseudomallei* biofilm growth dynamics and biofilm formation is inhibited by exogenous addition of sodium nitrate. Genomics analysis identified transposon insertional mutants in a predicted two-component system (*narX*/*narL*), a nitrate reductase (*narGH*), and a nitrate transporter (*narK*-*1*) required to sense nitrate and alter biofilm formation. Additionally, the results presented here show that exogenous nitrate reduces intracellular levels of the bacterial second messenger c-di-GMP. These results implicate the role of nitrate sensing in the regulation of a c-di-GMP phosphodiesterase and the corresponding effects on c-di-GMP levels and biofilm formation in *B. pseudomallei* 1026b.

## Introduction

Biofilms are dynamic natural communities of adherent bacteria encased in an extracellular polymeric matrix with signal-sensing systems primed to detect and respond to a variety of cues and environmental stimuli (O’Toole et al., 2000; McDougald et al., 2011). Biofilm formation in many pathogenic species is coordinated and positively regulated by the universal bacterial second messenger cyclic dimeric GMP (c-di-GMP), which facilitates the transition from free-swimming planktonic bacteria to sessile adherent communities (Tamayo et al., 2007). *Burkholderia pseudomallei* is a Gram-negative motile bacillus and saprophyte inhabiting tropical and subtropical soils and surface waters (Dance, 2000). This bacterium has been shown to persist in the environment as a pellicle (a biofilm that forms at the air-liquid interface) and as surface-associated biofilms on plants and in the rhizosphere of rice in endemic areas (Inglis and Sagripanti, 2006; Ramli et al., 2012; Prasertsincharoen et al., 2015). As an opportunistic pathogen, *B. pseudomallei* is acquired directly from the environment and can cause multiple types of infections in humans and a range of animal hosts (Dance, 2000). For a sapronotic disease agent such as *B. pseudomallei* (Kuris et al., 2014), it is important to understand the unique epidemiology related to its transition from the environment to establish persistent infections within susceptible hosts.

*Burkholderia pseudomallei* is found primarily within Southeast Asia and Northern Australia, where it is a prominent cause of bacterial pneumonia and sepsis, respectively (Hatcher et al., 2015). However, a recent report estimates the global distribution of *B. pseudomallei* is much larger than previously recognized and predicts environmental suitability across the tropics worldwide (Limmathurotsakul et al., 2016). *B. pseudomallei* infection results in melioidosis, a febrile illness whose clinical manifestations reflect the route of inoculation. Infection most commonly results from cutaneous exposure to contaminated soil or water, but also ingestion and respiratory inhalation (Wiersinga et al., 2012). Occupational exposure while working in rice fields is a primary source for acquisition of infection, but recreational activities such as gardening also expose individuals to *B. pseudomallei* in endemic areas (Limmathurotsakul et al., 2013; Kaestli et al., 2015). Anthropogenic manipulation of garden soils through irrigation and application of urea- and nitrate-rich fertilizers increases *B. pseudomallei* populations across different soil types (Kaestli et al., 2015). Nitrates are naturally occurring salts and byproducts of animal wastes, as well as artificial components of fertilizers, the use of which can lead to an imbalance in nutrient-limited soils (Kaestli et al., 2015). *B. pseudomallei* is associated with soil that contains elevated levels of nitrates and total nitrogen (Palasatien et al., 2008), anthrosol soil (Limmathurotsakul et al., 2016), as well as livestock and animal housing (Palasatien et al., 2008). This association with nitrogen levels is an apparent exception to the general negative association between *B. pseudomallei* and soil nutrient levels (Hantrakun et al., 2016).

Given the association of increased *B. pseudomallei* abundance in disturbed soils with elevated nitrate and organic materials, there is a critical need to understand the effects of exogenous nitrogen derivatives on *B. pseudomallei* physiology and epidemiology. Members of the *Burkholderia* genus, including the clinical isolate *B. pseudomallei* K96243, can grow in anaerobic conditions using nitrate as an alternative terminal electron acceptor, much like other facultative anaerobic bacteria (Yabuuchi et al., 1992; Hamad et al., 2011). The effects of anaerobic growth using nitrate on the physiology of *B. pseudomallei* biofilms remains largely undetermined. Interestingly, genes associated with nitrate reduction are implicated in biofilm formation in *B. thailandensis*, yet a mechanism of action has not been determined (Andreae et al., 2014). Nitrate sensing and metabolism have also been shown to influence biofilm formation and virulence in *Pseudomonas aeruginosa* (Van Alst et al., 2007; Zemke et al., 2013) and nitrite is known to inhibit biofilm formation in *Staphylococcus aureus* (Schlag et al., 2007). Additionally, nitrite metabolism is implicated in modulating antibiotic susceptibility in *P. aeruginosa* (Zemke et al., 2014, 2015).

To address the effects of exogenous nitrate on *B. pseudomallei* biofilms, transposon insertional mutants in predicted nitrate metabolism, regulatory, and sensory genes were identified and assessed for their ability to form biofilms in the presence of sodium nitrate. Inhibition of biofilm formation by sodium nitrate was not observed in five of the 21 transposon mutants tested: Bp1026b_I1013::T24 (*narL)*, Bp1026b_I1014::T24 (*narX*), Bp1026b_I1017::T24 (*narH-1*), Bp1026b_I1018::T24 (*narG-1*), and Bp1026b_I1020::T24 (*nark-1*). The dissimilatory nitrate reductase encoded by the *narGHJI* operon, which is conserved among both facultative and obligate anaerobes, is a membrane-bound enzyme complex comprised of four subunits responsible for nitrate reduction in the absence of oxygen (Payne, 1981; Sodergren et al., 1988). The *narX/narL* system is instrumental in nitrate metabolism and regulation as it has been shown to activate nitrate-specific enzymes and repress unnecessary anaerobic respiration genes in response to exogenous nitrate (Egan and Stewart, 1991; Goh et al., 2005). In this study, we show that the alpha and beta subunits of the major nitrate reductase *narGHJI-1* along with the *narX/narL* two-component regulatory system and the nitrate/nitrite transporter *narK-1* are responsible for biofilm inhibition based on nitrate availability.

These data demonstrate that nitrate sensory cascades and metabolism are linked to biofilm growth dynamics in *B. pseudomallei* 1026b. Moreover, we show a significant reduction in c-di-GMP levels in *B. pseudomallei* grown in the presence of nitrate. We hypothesize that nitrate sensing and nitrate-dependent anaerobic respiration promote a planktonic lifestyle transition in *B. pseudomallei* that is ultimately a result of depletion of intracellular levels of c-di-GMP and a corresponding decrease in biofilm formation.

## Materials and Methods

### Bacterial Strains and Growth Conditions

All experiments were performed in biosafety level 3 (BSL-3) facilities within the Regional Biocontainment Laboratory at Colorado State University with approvals from the Center for Disease Control and Prevention and the Colorado State University Institutional Biosafety Committee. *B. pseudomallei* strain 1026b, a clinical isolate from a human case of septicemic melioidosis in Thailand (Deshazer et al., 1997), was used in this study. Transposon (T24) mutant strains used in this study were generated during the production of a comprehensive two-allele sequence defined transposon mutant library (manuscript in preparation). T24 is a Tn*5*-derived transposon containing a kanamycin resistance selection marker approved for use in *B. pseudomallei* that was constructed in Colin Manoil’s laboratory at the University of Washington (manuscript in preparation). Briefly, the two-allele library was assembled via random transposon insertion into gene loci spanning both chromosomes of *B. pseudomallei* 1026b, resulting in insertions into 4,931 of 6,070 (81.2%) total predicted genes. In total, the library contains two copies of 4,415 genetic mutants that were re-sequenced. All transposon insertion mutants were verified by additional sequencing for these studies. Bacterial strains were grown in Lysogeny Broth (LB) media consisting of 10 g/L tryptone (Fisher Scientific), 5 g/L yeast extract (Becton, Dickinson and Company), and 5 g/L NaCl (Fisher Scientific) as previously described (Plumley et al., 2017). For experiments involving media with sodium nitrate or sodium nitrite, LB was supplemented with a final concentration of 10 mM sodium nitrate (Sigma) or 10 mM sodium nitrite (Matheson, Coleman & Bell) unless otherwise stated. Transposon mutants were grown in LB with 300 μg/mL kanamycin (Gold Biotechnology).

### Identification of Putative Nitrogen Metabolism Genes in *B. pseudomallei* 1026b

A combination of open-access genomic databases were used to assess the comparative identity of genetic orthologs in closely related *Burkholderia* spp. as well as *P*. *aeruginosa* strain PAO1. Predicted nucleotide sequences in the *B. pseudomallei* 1026b (tax id: 884204) genome for chromosomes I and II (accession numbers NC_017831.1 and NC_017832.1, respectively), were acquired from the NCBI GenBank database using the BLASTN program under default parameters to identify sequences similar to the PAO1 reference genome (GenBank assembly accession: GCA_000414275.1). Functional nitrogen metabolism pathways were evaluated using the Kyoto Encyclopedia of Genes and Genomes (KEGG) (Ogata et al., 1999). Further comparative genomic analyses, as well as gene annotations and locus positions were deduced using the Burkholderia Genome Database (Winsor et al., 2008) in conjunction with the Pseudomonas Genome Database (Winsor et al., 2015). Sequence comparison among nitrogen metabolism genes was analyzed with the following: *B. pseudomallei* K96243 (tax id: 272560), *B. thailandensis* E264 (tax id: 271848), and *P. aeruginosa* PAO1 (tax id: 208964). Percent identity between genetic orthologs was calculated using the Multiple Sequence Comparison by Log-Expectation (MUSCLE) tool provided by the European Bioinformatics Institute (EMBL-EBI), which calculates a percent identity matrix using Clustal 2.1 (Edgar, 2004). Gene names and functional descriptions were not entirely available for *B. pseudomallei* 1026b, and in such cases, were assigned based on consensus among the better annotated species detailed above.

### Comparative Analysis of Predicted Nitrogen Metabolism Clusters

Sequence analyses for the predicted nitrogen metabolism clusters encoded on chromosome I and II from the sequenced genome of *B. pseudomallei* 1026b (tax id: 884204) were visualized using open-source bioinformatics platforms and genomic sequence databases. We used BLASTN (BLAST, NCBI) under default parameters in conjunction with the Burkholderia Orthologous Groups cataloging system from the Burkholderia Genome Database^1^ to identify homologous regions. Sequences for *B. pseudomallei* 1026b chromosome I (accession number: NC_017831.1) and chromosome II (accession number: NC_017832.1) were downloaded from the GenBank database (NCBI, NIH). Sequence fragments were extracted with Geneious version 7.1.7^2^ (Kearse et al., 2012) and visualized with EasyFig version 2.23 (Sullivan et al., 2011) using Python version 2.7^3^. To calculate homology between input sequences, command line parameters for EasyFig included a minimum identity cut-off for BLASTN of 0.60 and a threshold *E*-value of 1E-3.

### Pellicle Biofilm Assays

Overnight cultures were grown in LB medium (kanamycin as indicated for transposon mutants) and sub-cultured 1:50 in 2 mL LB with or without 10 mM sodium nitrate. Pellicles were grown in 17 mm × 100 mm polystyrene tubes (#1485-2810, USA Scientific) at 37°C for 24 h before incubation at room temperature for 14 days without disturbance then photographed.

### Static Biofilm Assays

Overnight cultures were grown in LB and diluted to OD_600_ of 0.1 in LB with or without 10 mM sodium nitrate. Experiments using sodium nitrite were performed in the same manner as those using sodium nitrate. Gradient concentrations {0, 1, 2, 3, 5, 8, 10, 20, 50, and 100 mM} of sodium nitrate, sodium nitrite, or sodium chloride in LB were evaluated as indicated. One hundred microliters was added, in replicates of six, to individual wells of a 96-well polystyrene plate (Nunc^TM^ Microwell^TM^ 96-well microplates #243656, Thermo Scientific) and incubated at 37°C for 24 h. Static biofilms were processed as previously described (Plumley et al., 2017). Briefly, bacterial supernatant was removed from the biofilms and wells were washed once with PBS to remove non-adherent cells. The wells were stained with 0.05% crystal violet (Sigma–Aldrich, St. Louis, MO, United States) and incubated at room temperature for 15 min. Stain was removed and wells were washed again with PBS. Adherent crystal violet was solubilized by addition of 95% EtOH and incubated for 30 min. The solubilized dye was transferred to a new 96-well polystyrene plate and the absorbance was measured at OD_600_ on a Synergy HT plate reader (BioTek Instruments, Winooski, VT, United States). Significance was calculated with an unpaired Student’s *t*-test using the Holm-Sidak method to account for multiple comparisons.

### Complementation of Bp1026b_I1013::T24 (*narL*) and Bp1026b_I1014::T24 (*narX*) Mutants

Complementation constructs were designed for conditional expression of re-integrated genes via IPTG induction from the *tac* promoter, following select-agent-compliant methods for genetic modification in *B. pseudomallei* (Choi et al., 2008; Plumley et al., 2017). The full-length sequences of Bp1026b_I1013 (*narL*) and Bp1026b_I1014 (*narX*) were amplified using the following PCR primer pairs: (5′-NNNCCCGGGAGGAGGAT ATTCATGACCATACGGGTACTGTT-3′) and (5′-NNNAAGC TTTTATGCCTCGGCCGGATGCG-3′) for Bp1026b_I1013, and (5′-NNNCCCGGGAGGAGGATATTCATGGCTCCCGCC CTCCCCGA-3′) and (5′-NNNAAGCTTCTATGCCGCCTGTC GCGCGT-3′) for Bp1026b_I1014. Cloned genes were ligated into pUC18T-mini-Tn*7*T-Km-LAC, which uses the *tac* promoter from *Escherichia coli* to drive gene expression (Rholl et al., 2011). 5 mM IPTG was used to induce expression from the *tac* promoter. 5 mM NaNO_3_ was used to stimulate biofilm inhibition.

### Growth Curves

Overnight cultures were grown in LB and diluted to OD_600_ of 0.1 in LB supplemented with increasing concentrations of sodium nitrate or sodium chloride as indicated. Sodium chloride was supplemented in addition to the original 170 mM NaCl concentration in LB. Each experimental growth condition was repeated in six replicates. One hundred microliters of each culture was added to a 96-well polystyrene plate (Nunc^TM^ Microwell^TM^ 96-well microplates #243656, Thermo Scientific) in triplicate. Plates were incubated at 37°C shaking aerobically at 250 rpm for 48 h. Absorbance (OD_600_) was measured hourly on a Synergy HT plate reader (BioTek Instruments).

### Extraction and Quantification of c-di-GMP

Nucleotide extraction methods using formic acid were adapted from Massie et al. (2012). Overnight cultures of *B. pseudomallei* 1026b were grown in LB at 37°C shaking (250 rpm). Cultures were diluted 1:50 in 4 mL of M9 Salts minimal medium with or without 10 mM sodium nitrate supplemented and grown statically at 37°C for 18 h in six-well Costar polystyrene plates (#3516, Corning). Cells from 1 mL culture including the pellicle biofilm were collected and resuspended in 100 μL of cold 40% (vol/vol) acetonitrile/40% (vol/vol) methanol/20% (vol/vol) LC-MS-grade water/1% formic acid. An internal control of 2-chloro-adenosine-5′-*O*-monophosphate (2-Cl-5′-AMP, Axxora, LLC) was used at a final concentration of 100 nM (Irie and Parsek, 2014). The buffer, cells, and matrix were incubated at 65°C for 10 min and then immediately transferred to -20°C for 30 min to ensure complete lysis. Samples were then centrifuged at 16,000 × *g* at 4°C for 5 min and the supernatant fraction containing c-di-GMP was collected and neutralized with 20 μL 1 M KOH, followed by another spin at 16,000 × *g* at 4°C for 5 min. A calibration curve using known standards of chemically synthesized c-di-GMP (Axxora, LLC) and 2-Cl-5′-AMP was generated and extracted in identical conditions to experimental samples. Absolute quantification of c-di-GMP was calculated using the linear regression equation generated from known standards and peak areas normalized to the internal standard. Analysis was performed on an Acquity M-Class UPLC (Waters) coupled to a Xevo TQ-S triple quadrupole mass spectrometer (Waters) for LC-MS/MS. A Waters Atlantis dC18 stationary phase column (300 μm × 150 mm, 3.0 μm) was used for chromatographic separations. Protein pellets from the initial cell collection were analyzed for total protein concentration using the Pierce 660 nm Protein Assay (Thermo Scientific) for normalization of the absolute concentration of c-di-GMP. Nucleotide extraction experiments were repeated on two different days using four biological replicate cultures with three technical replicates each. Statistical significance was determined using an unpaired Student’s *t*-test in GraphPad Prism.

### RNA Isolation and Quantitative Real-Time PCR

*Burkholderia pseudomallei* 1026b was grown statically in M9 salts minimal media with or without 10 mM sodium nitrate supplemented at 37°C in six-well Costar polystyrene plates (Corning). After 18 h of static growth, 1 mL of culture was collected and cells were centrifuged at 12,000 × *g* for 2 min at 4°C. Bacterial pellets were resuspended in 350 μL RNAprotect Bacteria Reagent (Qiagen) before centrifuging at 5,000 × *g* for 10 min at 4°C. Bacterial pellets were resuspended in 1 mL QIAzol Lysis Reagent (Qiagen), incubated for 5 min at room temperature, and transferred to screwcap tubes containing 250 μL of 0.1 mm glass disruption beads (RPI Corp.) on ice. Cells were lysed using a TissueLyser II homogenizer (Qiagen) using three rounds of 60 s pulses at 30 Hz with 60 s on ice between each lysis round. After lysis, the cell mixture was incubated at room temperature for 5 min before adding 200 μL chloroform and vortexing for 5 s. Samples were incubated again at room temperature for 5 min before centrifugation at 13,000 × *g* for 10 min. The aqueous phase was collected and RNA was purified with an RNeasy Mini Kit (Qiagen) using the protocol recommended by the manufacturer. RNA samples were treated with two rounds of TURBO DNase (Ambion) before cDNA was synthesized using the Transcriptor First Strand cDNA Synthesis Kit (Roche). qRT-PCR was performed on a LightCycler 480 System with SYBR Green I Master (Roche) using 10 ng total cDNA and the following cycling conditions: pre-incubation at 95°C for 10 min; three-step amplification at 95°C for 10 s, 55°C for 15 s, 72°C for 15 s; melting curve at 95°C for 5 s, 65°C for 1 min, and 97°C for continuous acquisitions per 5°C. Primers were designed using the PrimerQuest tool (IDT DNA Technologies). Previously published 23S rRNA primers were used for normalization (Mima and Schweizer, 2010). All sets of primers were validated for amplification efficiency in a standard curve. The primer set used for *cdpA* was as follows: forward (5′-AAGCTGGCTGGAGCAAA-3′) and reverse (5′-GCAGATAGTCGCGGTGATAA-3′). The primer set used for Bp1026b_II2523 was as follows: forward (5′-GCAAGATCGAGAGCGTGTT-3′) and reverse (5′-GTGATCGAAGCGGAACAGATAC-3′). The primer set used for Bp1026b_ II0885 was as follows: forward (5′-CTGCACCTACCGCTTCTT-3′) and reverse (5′-AAGCACAGCGAGAAGTAGTC-3′). The primer set used for Bp1026b_I3148 was as follows: forward (5′-CGCTGCATCTGGAGAACTT-3′) and reverse (5′-CCTGAAACGGATCGGTGAG-3′). Transcript abundance was measured using the Pfaffl method (Pfaffl, 2001), taking into account primer amplification efficiency, including three independent biological samples performed in technical triplicates.

## Results

### Nitrate Inhibits *B. pseudomallei* Biofilm Formation

**FIGURE 1:**
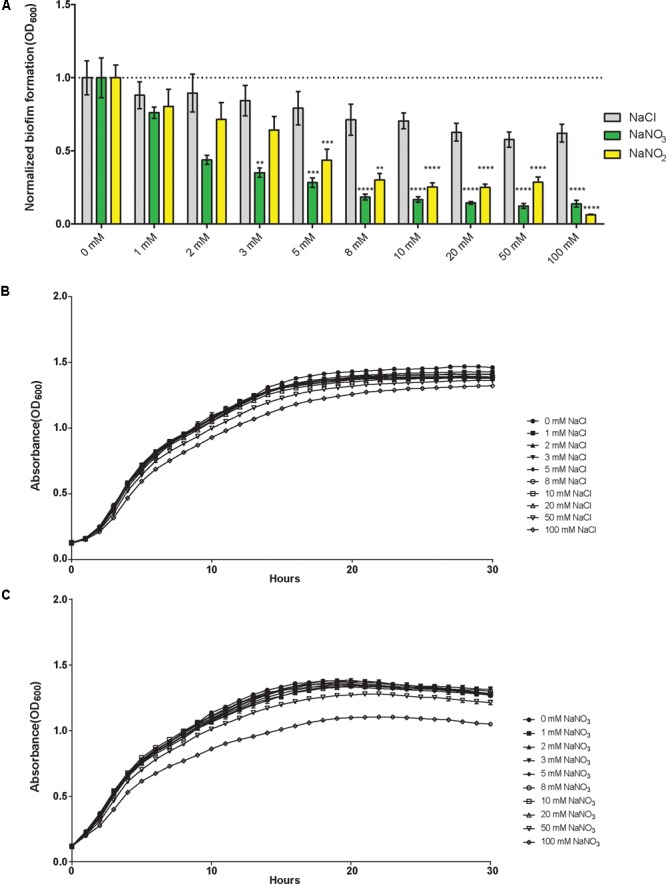
Sodium nitrate and sodium nitrite, but not sodium chloride, inhibit biofilm formation in *Burkholderia pseudomallei* 1026b. **(A)** Biofilm inhibition was assessed for static biofilm cultures growing at 37°C in the presence of sodium chloride, sodium nitrate, or sodium nitrite at 1, 2, 3, 5, 8, 10, 20, 50, and 100 mM. All experiments were performed with wild-type *B. pseudomallei* 1026b and inhibition was evaluated relative to wild-type biofilm formation in LB without additional salts added. Asterisks indicate a significant difference (^∗∗^*p* < 0.01, ^∗∗∗^*p* < 0.001, ^∗∗∗∗^*p* < 0.0001) calculated with an unpaired Student’s *t*-test using the Holm-Sidak method to account for multiple comparisons (*n* = 12). Growth curves were generated from cultures grown with shaking at 37°C in the presence of either sodium chloride **(B)** or sodium nitrate **(C)** using concentrations as indicated for the biofilm inhibition assay. Absorbance (OD_600_) measurements were taken hourly for 30 continuous hours.

While the effectors of biofilm dispersal and inhibition are extensive and diverse, nitrogenous compounds have been shown to modulate biofilm formation in both Gram-negative and Gram-positive bacteria (Schlag et al., 2007; Van Alst et al., 2007; Zemke et al., 2014). Sodium nitrate (NaNO_3_), a naturally occurring salt compound and a synthetic agricultural additive, inhibits biofilm formation in a dose-dependent manner (**Figure 1A**). Likewise, sodium nitrite also inhibits biofilm formation following a similar trend, although the effect is not as robust initially (**Figure 1A**). To assess the impact of nitrate/nitrite on biofilm formation, wild-type *B. pseudomallei* 1026b was grown statically for 24 h with increasing concentrations of sodium nitrate or sodium nitrite (**Figure 1A**). Biofilm inhibition effects were first noted at 1 mM while the most significant biofilm inhibition occurred with the addition of 10 mM sodium nitrate or 10 mM sodium nitrite. There were no additional inhibitory effects on biofilm formation with treatment concentrations greater than 10 mM NaNO_3_. As such, concentrations of 10 mM NaNO_3_ were used for all subsequent experiments. To evaluate the effects of sodium concentration on biofilm formation, experiments with only sodium chloride (NaCl) indicated that biofilm inhibition was not significantly altered during biofilm formation under the same conditions (**Figure 1A**). Viability was also tested by measuring growth kinetics in media supplemented with NaCl (**Figure 1B**) and NaNO_3_ (**Figure 1C**). Growth inhibition was observed only when cells were grown in media supplemented with 100 mM of NaNO_3_ and 100 mM of NaCl, concentrations that are well beyond those used in this study. No significant differences in growth dynamics were observed at 10 mM NaNO_3_ suggesting that nitrate dosing at this concentration does not affect cell viability. These results suggest that nitrate sensing and metabolism, and not sodium, is responsible for biofilm inhibition in *B. pseudomallei* 1026b and the observed inhibition is not the result of decreased cellular viability.

### Bioinformatics Analysis of Predicted Nitrogen Metabolism Genes

Bioinformatics analyses identified 25 *B. pseudomallei* 1026b genes predicted to be involved in nitrogen metabolism (**Table 1**), of which 21 were included in this study based on the availability from a two-allele sequence defined transposon library. The genes included in this study were selected based on sequence homology among closely related *Burkholderia* spp. to *P. aeruginosa* PAO1, which has an extensively annotated reference genome that has been biochemically validated for the nitrogen metabolism genes. Within the Proteobacteria phylum, the *β-proteobacteria*, which contains the genus *Burkholderia*, is closely related to the genus *Pseudomonas*, which is a *γ-proteobacteria* (Estrada-De Los Santos et al., 2013), thus inter-genus comparisons described in this study will provide insights into the functions among these conserved nitrogen metabolism proteins. While *B. pseudomallei* 1026b has many genes predicted to encode enzymes in the denitrification pathway, there is a need for more comprehensive functional annotation in this relatively new clinically isolated strain. Therefore, nucleotide sequence identity was analyzed across three *Burkholderia* species in relation to *P. aeruginosa* PAO1 (**Table 1** and Supplementary Table 1).

**Table 1:** Predicted nitrate metabolism genes identified in this study.

Name and predicted function	Putative annotation^a^	*B. pseudomallei*^b^	*P. aeruginosa*	Nucleotide similarity^c^(%)
DNA-binding response regulator	*narL*	Bp1026b_I1013	PA3879	65.12
Nitrate/nitrite sensor protein	*narX*	Bp1026b_I1014	PA3878	65.00
Nitrate reductase (1)				
Gamma subunit	*narI-1*	Bp1026b_I1015	PA3872	64.50
Delta subunit	*narJ-1*	Bp1026b_I1016	PA3873	72.54
Beta subunit	*narH-1*	Bp1026b_I1017	PA3874	75.88
Alpha subunit	*narG-1*	Bp1026b_I1018	PA3875	75.20
Nitrate/nitrite transporter	*narK-2*^d^	Bp1026b_I1019	PA3876	73.90
Nitrate/nitrite transporter	*narK-1*^d^	Bp1026b_I1020	PA3877	69.76
Nitric oxide reductase	*norB*	Bp1026b_I0974	PA0524	68.37
Nitrous oxide reductase	*nosZ*	Bp1026b_I1546	PA3392	68.47
Assimilatory nitrite reductase (NAD(P)H) (1)				
Large subunit	*nirB-1*	Bp1026b_I2984	PA1781	76.39
Small subunit	*nirD-1*	Bp1026b_I2985	PA1780	69.23
Assimilatory nitrate reductase (1)				
Molybdopterin oxidoreductase family protein	*nasA-1*^e^	Bp1026b_I2986	PA1779	67.45
Nitrate/nitrite transporter	*narK*	Bp1026b_II1220	PA3870	59.89
Nitrate reductase (2)				
Gamma subunit	*narI-2*	Bp1026b_II1222	PA3872	65.45
Delta subunit	*narJ-2*	Bp1026b_II1223	PA3873	65.60
Beta subunit	*narH-2*	Bp1026b_II1224	PA3874	78.55
Alpha subunit	*narG-2*	Bp1026b_II1225	PA3875	74.14
Assimilatory nitrate reductase (2)				
Large subunit	*nasA-2*	Bp1026b_II1316	PA1779	66.77
Assimilatory nitrite reductase (NAD(P)H) (2)				
Small subunit	*nirD-2*	Bp1026b_II1317	PA1780	64.53
Large subunit	*nirB-2*	Bp1026b_II1318	PA1781	62.74
Nitrate regulator	*nasT*^d^	Bp1026b_II1319	PA1783	61.92
Nitrate transport ATP-binding protein	*nasS*	Bp1026b_II1322	PA1786	68.54
Nitrite reductases				
Multicopper oxidase domain-containing protein	*nirS*^d^	Bp1026b_II1540	PA0519	60.60
Anaerobically induced outer membrane protein	*nirK*^f^	Bp1026b_II1580	N/A	N/A

*^a^Gene annotations determined using the Burkholderia Genome Database.*

*^b^Locus ID determined using GenBank Database (NCBI – http://www.ncbi.nlm.nih.gov/) in conjunction with the Burkholderia Genome Database.*

*^c^Percent identity determined using MUSCLE (Multiple Sequence Comparison by Log-Expectation – http://www.ebi.ac.uk/).*

*^d^No clear annotations in *Burkholderia* spp.; Annotation from the Pseudomonas Genome Database.*

*^e^BP1026_I2986 and its homolog BPSL0510 (see Supplementary Material) are predicted orthologs of *Pseudomonas aeruginosa* assimilatory nitrate reductase NasA [see Andreae et al., 2014].*

*^f^Gene specific to *Burkholderia* spp.*

Orthologs of the denitrification enzymes in *B. pseudomallei* 1026b presented here share 99–100% nucleotide identity to the fully sequenced genome of *B. pseudomallei* K96243, 90–97% nucleotide identity to *B. thailandensis* E264, and 60–78% nucleotide identity to the more distant relative *P. aeruginosa* PAO1 (Supplementary Table 1). This underscores the highly conserved nature of the genes constituting the essential denitrification pathway among these soil-dwelling bacteria. Interestingly, bioinformatics analysis revealed duplications of specific genes implicated in the denitrification pathway in *B. pseudomallei* 1026b, which is consistent with the predicted genetic supplementation and metabolic redundancy of *B. pseudomallei* (Holden et al., 2004). The major nitrate reductases are encoded on chromosome I at Bp1026b_I1015 – Bp1026b_I1018 and on chromosome II at Bp1026b_II1222 – Bp1026b_II1225, and share sequence homology of 69–79% (Supplementary Table 2). Additionally, the major nitrite reductase is encoded on chromosome I at Bp1026b_I2984 – Bp1026b_I2985 and on chromosome II at Bp1026b_II1317 – Bp1026b_II1318, sharing only 65% nucleotide identity (Supplementary Table 2).

Bioinformatics analyses also identified putative nitrogen metabolism genes with no clear annotations across *Burkholderia* spp. Therefore, Bp1026b_I1019, Bp1026b_I1020, Bp1026b_II1319, and Bp1026b_II1540 were assigned genetic annotations based on the *P. aeruginosa* PAO1 reference genome. Two genes were identified as assimilatory nitrate reductases (Bp1026b_I2986 and Bp1026b_II1316) based on sequence homology to PAO1; however, no annotated homologs were available throughout *Burkholderia* spp., thus the annotation was assigned to be consistent with *P. aeruginosa* PAO1. Lastly, Bp1026b_II1580, which is predicted to encode an anaerobically induced outer membrane nitrite reductase, was specific to *Burkholderia* spp. with no representative homolog in *P. aeruginosa*.

**FIGURE 2:**
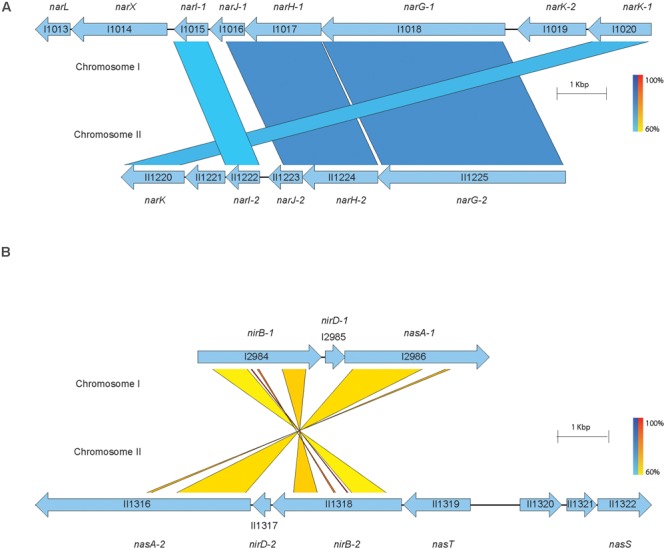
Predicted nitrogen metabolism gene clusters in *B. pseudomallei* 1026b. The putative nitrate and nitrite reductases, transporters, and nitrate-sensing gene clusters from the sequenced genome of *B. pseudomallei* 1026b are shown. A total of 21 loci spanning *B. pseudomallei* chromosomes I and II included in this study are illustrated here. Bp1026b_I1546, Bp1026b_II1540, and Bp1026b_II1580 were excluded. Coding sequences for the represented nitrate metabolism loci are depicted by arrows in relation to positive or negative strand orientation with open reading frames and intergenic regions illustrated to scale. The results of BLASTN annotations are depicted by color coded bars spanning gene loci across the two chromosomes with a minimum percent identity of 0.60 and a threshold *E*-value of 1E-3. Blue bars depict direct sequence homology and yellow-to-red bars depict homology amid sequence inversion, on a color density gradient indicating the percent homology. **(A)** The major predicted nitrate reductase *narGHJI* is encoded on chromosome I (Bp1026b_I1015 – 1018) as well as on chromosome II (Bp1026b_II1222 – 1225). Adjacent to the nitrate reductases are predicted nitrate transporters (Bp1026b_I1019, Bp1026b_I1020, Bp1026b_II1220) and the predicted two-component signaling system (Bp1026b_I1013 – I1014). **(B)** The major nitrite reductase *nirBD* is encoded on chromosome I (Bp1026b_I2984 – 2985) as well as on chromosome II (Bp1026b_II1317 – 1318). Adjacent to the nitrite reductase is the predicted assimilatory nitrate reductase *nasA* encoded by Bp1026b_I2986 on chromosome I and Bp1026b_II1316 on chromosome II. The predicted nitrate regulator, *nasT*, and nitrate transport ATP-binding loci, *nasS*, are encoded on chromosome II at Bp1026b_II1319 and Bp1026b_II1322, respectively.

Based on the information available from the previously described open-source genome databases and the genetic functional pathway predictions provided by the KEGG database, we mapped and compared the genetic arrangement of the predicted nitrogen metabolism enzymes across both chromosomes (**Figures 2A,B**). The *narX/narL* predicted two-component signaling system lies downstream and adjacent to the primary nitrate reductase on chromosome I, which is flanked by the cytoplasmic membrane-associated nitrate/nitrite transporters (**Figure 2A**). Another gene cluster of interest for these studies is the assimilatory nitrite reductase on chromosome I, with the adjacent molybdopterin oxidoreductase *nasA*, which is predicted to function as an assimilatory nitrate reductase (Ogawa et al., 1995) (**Figure 2B**). Interestingly, the nitrate metabolism gene clusters found on chromosome I are replicated in similar configurations on chromosome II. The relatively equal distribution of denitrification-associated loci across both *B. pseudomallei* chromosomes is not surprising considering that chromosome II is not an accessory genome and also encodes for essential and central metabolism genes (Holden et al., 2004); however, the predicted nitrate metabolism loci on chromosome II are not directly homologous to those on chromosome I. This disparity in sequence identity among similarly annotated gene loci with parallel genetic arrangements indicates that the nitrate metabolism clusters are not direct paralogs and may be required for specific metabolic functions based on extracellular cues.

### Five Genes within the Denitrification Pathway Contribute to Pellicle Biofilm Inhibition in the Presence of Exogenous Nitrate in *B. pseudomallei*

**FIGURE 3:**
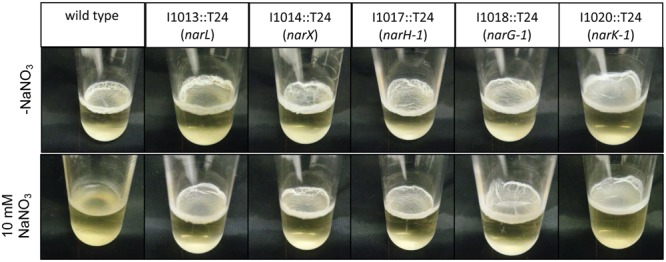
Five transposon insertions in the predicted nitrate metabolism pathway do not respond to inhibition of pellicle biofilm formation mediated by the addition of sodium nitrate. Wild-type *B. pseudomallei* 1026b forms a distinct pellicle biofilm when grown statically in liquid culture; however, it is unable to form a pellicle biofilm in the presence of 10 mM NaNO_3_. Transposon insertion mutants in the following genes {Bp1026b_I1013::T24 (*narL*), Bp1026b_I1014::T24 (*narX*), Bp1026b_I1017::T24 (*narH-1*), Bp1026b_I1018::T24 (*narG-1*), and Bp1026b_I1020::T24 (*nark-1*)} were identified that form pellicle biofilms growing statically in LB + 10 mM NaNO_3_. Pellicles were grown in 2 mL media at room temperature and photographed at 14 days.

We hypothesized that pellicle biofilms cannot form in the presence of exogenous nitrate under the conditions tested. Pellicle biofilms have been historically well characterized in the Gram-positive soil-dwelling bacterium *Bacillus subtilis* (Armitano et al., 2014), as well as pathogenic Gram-negative species such as *P. aeruginosa* (Cole et al., 1989) and *Burkholderia cenocepacia* (Fazli et al., 2013). *B. pseudomallei* forms pellicle biofilms in static cultures *in vitro* and at the surface-liquid interface near the root zone of plants. Wild-type and transposon insertional mutants of genes in the denitrification pathway were grown statically with or without sodium nitrate to assess the impact of nitrate on pellicle formation. The wild type did not form a pellicle biofilm in the presence of 10 mM NaNO_3_, while a robust pellicle biofilm was observed in LB without nitrate (**Figure 3**). Furthermore, pellicle formation was inhibited by sodium nitrate in 16 of 21 transposon insertional mutants (Supplementary Figure 1). Interestingly, five transposon insertional mutants in the denitrification pathway were resistant to inhibition of pellicle formation by exogenous nitrate (**Figure 3** and Supplementary Figure 1). Transposon insertions in *narL*, a DNA-binding response regulator, and *narX*, a nitrate sensor protein, which comprise a two-component signaling system regulated by nitrate, were resistant to pellicle inhibition by nitrate. Similarly, transposon mutants with insertions in *narG-1* and *narH-1*, which are predicted to be the major nitrate reductase alpha and beta subunits were also insensitive to inhibition of pellicle formation by nitrate. Interestingly, transposon mutations into *narG-1* and *narH-1* did not confer resistance to biofilm inhibition by sodium nitrite (Supplementary Figure 2), indicating that nitrite may inhibit biofilm formation independently of the major nitrate reductase. It is curious that only the cytoplasmic subunits of the major nitrate reductase were hit during our transposon screen for the biofilm inhibitory phenotype via sodium nitrate, an observation that may provide insights into the respiratory electron transfer pathway in *B. pseudomallei*. Additionally, it is worth noting that the membrane-anchored *narI-1* gamma subunit of the nitrate reductase was not available in our transposon mutant library, and as such, its relation to biofilm dynamics remains uncharacterized. We identified a fifth transposon mutant with an insertion in *narK-1*, encoding a predicted nitrate/nitrite transporter that was also insensitive to pellicle biofilm inhibition by nitrate. These results suggest that this genetic quintet, Bp1026b_I1013 (*narL*), Bp1026b_I1014 (*narX*), Bp1026b_I1017 (*narH-1*), Bp1026b_I1018 (*narG-1*), Bp1026b_I1020 (*nark-1*), contributes to the regulation and formation of *B. pseudomallei* biofilms when exogenous nitrate is encountered.

### Quantification of Biofilm Formation

**FIGURE 4:**
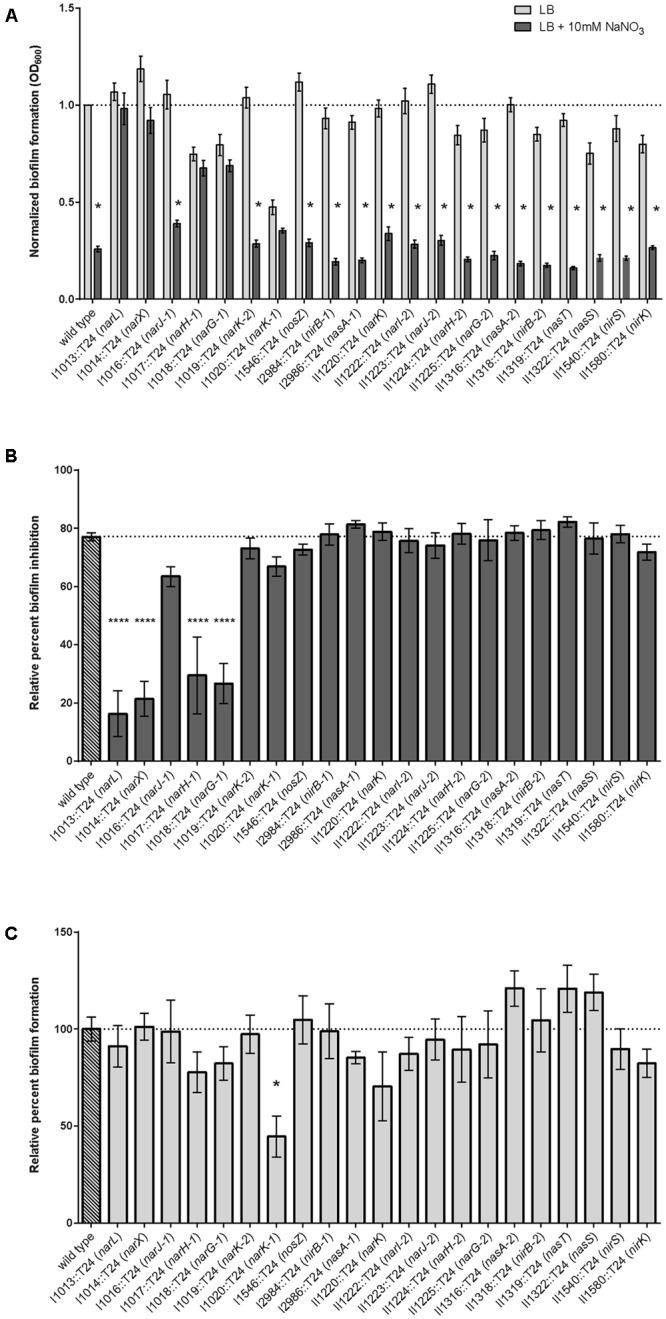
Quantitative evaluation of relative biofilm formation and biofilm inhibition in the presence and absence of sodium nitrate. **(A)** Biofilm formation was evaluated when grown statically in the presence of 10 mM NaNO_3_. Biofilm formation of the wild-type and 16 of the 21 transposon mutants identified in this study were inhibited by treatment with NaNO_3_, while five mutants {Bp1026b_I1013::T24 (*narL)*, Bp1026b_I1014::T24 (*narX*), Bp1026b_I1017::T24 (*narH-1*), Bp1026b_I1018::T24 (*narG-1*), and Bp1026b_I1020::T24 (*nark-1*)} were not inhibited by treatment with NaNO_3_. Bars are representative of the means for individually normalized values for biofilm formation relative to the wild type. Asterisks indicate a significant difference (*p* < 0.0001) calculated with an unpaired Student’s *t*-test using the Holm-Sidak method to account for multiple comparisons (*n* = 12). **(B)** Percent biofilm inhibition for all mutants was calculated relative to inhibition of the wild type in the presence of 10 mM NaNO_3_. Bars are representative of the means for each mutant relative to the mean for the wild type. **(C)** Biofilm formation for all mutants was calculated relative to wild type biofilm formation in LB medium. Bars are representative of the means for each mutant relative to the mean for the wild type.

Biofilm inhibition by nitrate was assessed in the same array of transposon insertion mutants using the quantitative biofilm growth method (O’Toole and Kolter, 1998) to calculate results from static biofilm assays. In conjunction with the direct observation of pellicle formation, the static biofilm assay provides quantitative analysis on the biofilm-forming capabilities for each strain in this study. Biofilm formation was significantly abolished with nitrate treatment in 16 of the 21 transposon mutants; however, biofilm formation of transposon mutants in *narL, narX, narG-1, narH-1*, and *narK-1* was not significantly altered in the presence of nitrate (**Figure 4A**). These results corroborated the qualitative observations of the pellicle biofilm assay, as the same five mutants were similarly resistant to the biofilm inhibitory effects of nitrate in both assays. Planktonic growth for *narL, narX, narG-1, narH-1*, and *narK-1* was similar when compared to wild-type *B. pseudomallei* 1026b (Supplementary Figure 3).

To further assess the effects of nitrate metabolism genes on biofilm inhibition, we calculated the percent of relative biofilm inhibition for all mutant strains in this study (**Figure 4B**). While wild-type biofilm formation was reduced by nearly 80% when sodium nitrate is encountered, transposon insertions in *narL, narX, narG-1*, and *narH*-*1* did not respond to the inhibitory effects of treatment with sodium nitrate. The decrease in the percentage of relative biofilm formation for the transposon-inactivated *narK-1* mutant (**Figure 4C**) indicates that this mutant is affected by nitrate more so than the *narL, narX, narG-1*, and *narH-1* mutants, and is also reflective of a fundamental biofilm-forming defect in the *narK-1* mutant. Taken together, these results provide quantitative measurements of biofilm inhibition and formation in the presence of exogenous nitrate, and support our conclusions regarding the role of these functionally inactivated genetic loci as observed in the pellicle biofilm assay.

**FIGURE 5:**
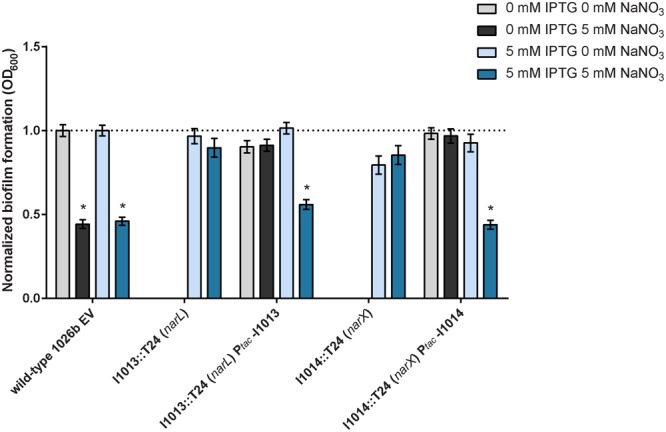
Complementation of the I1013::T24 (*narL*) and I1014::T24 (*narX*) mutants that are resistant to the inhibitory effects of exogenous nitrate. Mutants of the predicted nitrate-sensing two-component system *narX*/*narL* were complemented with full-length Bp1026b_I1013 (*narL*) and Bp1026b_I1014 (*narX*). The genes were re-introduced in the T24-mutant background at a neutral site and expression was conditionally induced using 5 mM IPTG. Biofilm formation was assessed in the presence and absence of 5 mM NaNO_3_. Asterisks indicate a significant difference (*p* < 0.0001) calculated with an unpaired Student’s *t*-test.

To confirm that the transposon mutant insertions identified in the T24-mutagenesis screen were causal to the observed phenotypes, we complemented I1013::T24 (*narL*) and I1014::T24 (*narX*) with full-length genes which were conditionally expressed with IPTG induction. The resulting constructs, Bp1026b_I1013::T24 P_tac_-I1013 (*narL*) and Bp1026b_I1014::T24 P_tac_-I1014 (*narX*) produced similar amounts of biofilm as the wild-type empty-vector (EV) control strain in the absence of NaNO_3_ (**Figure 5**). Conditional expression of *narL* in Bp1026b_I1013::T24 P_tac_-I1013 and *narX* in Bp1026b_I1014::T24 P_tac_-I1014 resulted in an approximate 50% reduction in biofilm formation with the addition of 5 mM NaNO_3_ that was equivalent to the wild-type EV control strain (**Figure 5**). These data further support the hypothesis that the *narX/narL* two-component signaling system, which is predicted to respond to nitrate sensing, is implicated in biofilm growth dynamics in *B. pseudomallei*.

### Exogenous Nitrate Reduces Intracellular c-di-GMP in a Static Biofilm Assay

**FIGURE 6:**
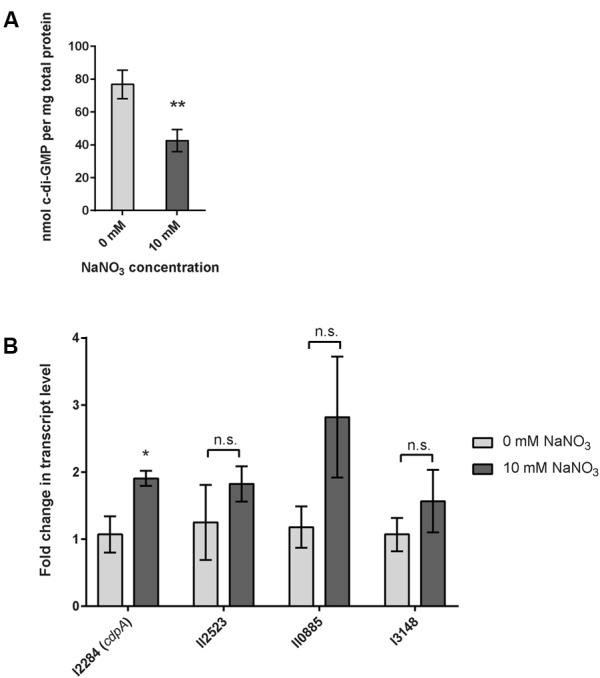
Quantification of cyclic dimeric GMP (c-di-GMP) in response to sodium nitrate and relative transcript abundance of *cdpA*, the major phosphodiesterase in *B. pseudomallei*. **(A)** The intracellular concentration of the bacterial second messenger c-di-GMP is significantly reduced in *B. pseudomallei* 1026b grown statically in nitrate-supplemented media. Error bars indicate standard error for four biological replicates extracted on different days. Statistical significance was determined using the Sidak-Bonferroni method across multiple *T*-tests (^∗∗^*p* < 0.01). **(B)** *cdpA* transcript level is upregulated nearly twofold in *B. pseudomallei* 1026b grown statically in nitrate-supplemented media. Three additional transcripts that encode for a phosphodiesterase (I3148), a diguanylate cyclase (II2523), and a composite diguanylate cyclase/phosphodiesterase (II0885) did not show statistical differences in the levels of transcript. Fold change in transcript level was calculated using the Pfaffl method considering primer amplification efficiency and normalized to the reference transcript for 23S rRNA. Statistical significance was determined using a one-tailed heteroscedastic Student’s *t*-test (^∗^*p* < 0.05).

Biofilm formation and dispersal mechanisms are ultimately dependent on the level of intracellular c-di-GMP in many pathogenic bacteria (Tamayo et al., 2007), thus we hypothesized that biofilm inhibition via exogenous nitrate sensing regulates c-di-GMP concentrations in *B. pseudomallei* 1026b. We analyzed the intracellular concentration of c-di-GMP via liquid chromatography tandem-mass spectrometry (LC-MS/MS) using a triple quadrupole mass spectrometer. The intracellular concentration of c-di-GMP in wild-type bacteria grown statically in minimal media with 10 mM sodium nitrate was reduced by 38% as compared to biofilms cultivated statically in the absence of nitrate (**Figure 6A**). For static biofilms cultivated without sodium nitrate, we observed an average concentration of 80.46 nmol c-di-GMP per mg of total protein and for biofilms cultivated in the presence of 10 mM nitrate the average concentration was 50.19 nmol c-di-GMP per mg total protein (**Figure 6A**). These results suggest that nitrate metabolism regulates intracellular c-di-GMP levels in *B. pseudomallei* 1026b biofilms.

### The Phosphodiesterase *cdpA* Is Upregulated in Response to Nitrate in a Static Biofilm Assay

In a simplified model of c-di-GMP regulation, a decrease in intracellular c-di-GMP levels suggests either decreased diguanylate cyclase activity or increased phosphodiesterase activity. We chose to evaluate the expression of previously described diguanylate cyclases and phosphodiesterases that are known to alter biofilm dynamics in *B. pseudomallei* (Lee et al., 2010; Plumley et al., 2017). C-di-GMP metabolism in bacteria, including *B. pseudomallei* 1026b, is classically mediated by diguanylate cyclases and phosphodiesterases that contain conserved GG(D/E)EF and EAL catalytic domains, respectively (Tamayo et al., 2007; Plumley et al., 2017). *B. pseudomallei* 1026b contains six predicted proteins with conserved EAL domains and five predicted GGDEF-EAL composite proteins (Plumley et al., 2017); however, only one of these predicted proteins, Bp1026b_I2284, has been shown to potentially function as a classical phosphodiesterase (Plumley et al., 2017). Bp1026b_I2284 (*cdpA*) is a previously described phosphodiesterase and known regulator of c-di-GMP levels in *B. pseudomallei* KHW (Lee et al., 2010). Recently, Bp1026b_I2284 (*cdpA*) has also been shown to contribute to motility and is therefore potentially implicated in reducing c-di-GMP levels in *B. pseudomallei* 1026b during planktonic growth (Plumley et al., 2017). Thus, we hypothesized that *cdpA* transcript expression is regulated during nitrate metabolism in *B. pseudomallei* 1026b, leading to a decrease in intracellular c-di-GMP and inhibition of biofilm formation.

During the transition between biofilm and planktonic states multiple phosphodiesterases and diguanylate cyclases may be expressed in a coordinated manner to alter the levels of c-di-GMP in response to stimulatory signals and conditions. To begin to address the complexity associated with c-di-GMP signaling during nitrate metabolism, we evaluated the transcription of previously identified phosphodiesterase and diguanylate cyclase genes (Plumley et al., 2017). Bp1026b_II0885, a composite protein that is similar to *cdpA*, was chosen for analysis based on the conservation of the EAL domain and GGDEF catalytic domains (Plumley et al., 2017). Bp1026b_I3148 was also chosen based on the high level of conservation of the EAL domain (Plumley et al., 2017). To evaluate the effects on diguanylate cyclase gene expression, we chose to analyze Bp1026b_II2523 based on its conserved GGEEF domain and its previously observed role in the thermoregulation of biofilm growth dynamics (Plumley et al., 2017).

To assess the impact of exogenous nitrate on the expression of c-di-GMP phosphodiesterases and diguanylate cyclases, wild-type *B. pseudomallei* 1026b was grown statically under biofilm-inducing conditions in minimal media with or without 10 mM NaNO_3_. The expression of *cdpA* was significantly higher, representing a nearly twofold increase in cells grown in 10 mM NaNO_3_-supplemented minimal media when normalized to 23S (**Figure 6B**). Interestingly, the expression values for the additional two transcripts that potentially encode c-di-GMP phosphodiesterases showed a similar trend to that of *cdpA*; however, these analyses lacked statistical significance (**Figure 6B**). The expression of Bp1026b_II2523, a putative diguanylate cyclase showed no significant response to nitrate, which indicates it may not be involved in the nitrate-dependent biofilm inhibition cascade under the conditions tested. Thus, while nitrate has a clear regulatory effect on *cdpA*, it is possible that other phosphodiesterases and diguanylate cyclases that remain to be tested also contribute to the intracellular concentration of c-di-GMP. Moreover we showed that *cdpA* is not required for nitrate- or nitrite-dependent biofilm inhibition (Supplementary Figure 4). Nonetheless, these results indicate that nitrate can potentially increase phosphodiesterase activity in *B. pseudomallei* 1026b and can explain the significant decrease in c-di-GMP concentration during growth under identical conditions. Taken together with the absolute quantification of c-di-GMP, these data support our hypothesis that exogenous nitrate inhibits *B. pseudomallei* 1026b biofilms by reducing the intracellular concentration of c-di-GMP.

## Discussion

Environmental acquisition of opportunistic pathogens such as *B. pseudomallei* requires a lifestyle transition that prompts a shift from the natural reservoir to establish infections in susceptible human and animal hosts. Biofilm communities serve as a predominant natural habitat for the saprophytic *B. pseudomallei*, which colonizes and persists in the rhizosphere of plants in wet soils of endemic areas (Prasertsincharoen et al., 2015), thus efforts to better understand the dissemination from this environment would serve to mitigate public health risks related to the emerging infectious disease melioidosis. The particular effectors that serve as biofilm inhibition signals and dispersal cues for *B. pseudomallei* are poorly understood, although previous studies link anthropogenic disturbances to increased bacterial presence in endemic areas (Kaestli et al., 2009; Limmathurotsakul et al., 2013, 2016). Notably, and of particular interest to our present study, are the observations by Kaestli et al. (2015) that addition of nitrate-rich fertilizer increased *B. pseudomallei* bacterial loads across a variety of soils. Since nitrate (NO_3_^-^) and nitrite (NO_2_^-^) can serve as terminal electron acceptors for anaerobic respiration in denitrifying bacteria (Payne, 1981) and the *Burkholderia* genera are capable of denitrification (Kaestli et al., 2015), we tested the hypothesis that nitrate inhibits biofilm formation in *B. pseudomallei* 1026b.

The results presented in this study indicate that exogenous addition of nitrate, and to a lesser extent, nitrite, inhibit biofilm formation. While both nitrate and nitrite have been shown to repress biofilm formation in *P. aeruginosa* (Van Alst et al., 2007) and *S. aureus* (Schlag et al., 2007), respectively, this is the first study to address the effects of nitrate on *B. pseudomallei* biofilm formation. We identified a total of 21 genes, which were predicted to encode nitrate metabolism enzymes in the denitrification pathway, of which five transposon mutants no longer responded to biofilm inhibition by nitrate. This analysis identified the predicted two-component signaling system *narX*/*narL*, and implicates this conserved regulatory system in nitrate metabolism as well as control of biofilm growth dynamics in *B. pseudomallei*. The nitrate-sensing two-component system, which is conserved among aerobes and anaerobes alike, mediates gene expression when *narX* senses environmental nitrate, thereby stimulating a phosphorylation cascade that causes *narL* to bind and regulate DNA transcription (Walker and DeMoss, 1993). Thus, the *narX/narL* system acts as a primary sensor of exogenous nitrate, and our observations implicate this system in the genetic control of biofilm formation. Given the genetic arrangement of this two-component system adjacent to the predicted major nitrate reductase on chromosome I (**Figure 2**), future studies will address whether the DNA-binding and regulatory effects of NarL are targeted to the upstream *narGHJI*-*1* operon.

Our results also indicate that two subunits in the major dissimilatory nitrate reductase *narGHJI-1*, which is tasked with reducing nitrate to nitrite inside the bacterial cell, contributes to exogenous nitrate sensing and control of biofilm formation when nitrate is present. Biofilm formation is also inhibited by nitrite (**Figure 1A**), and although this genetic mechanism has not been investigated in the present study, our results indicate that this phenotype occurs independently of the alpha and beta subunits of the primary nitrate reductase *narGH-1* (Supplementary Figure 2). Additionally, biofilm formation of a transposon mutant insertion in one of the four nitrate/nitrite transporter genes, *narK-1*, was no longer inhibited in the presence of nitrate. These results suggest that the five genes *narL, narX, narG-1, narH-1*, and *narK-1* are implicated in mediating the biofilm growth dynamics of *B. pseudomallei* when exogenous nitrate is encountered in the environment. Given that the *narX/narL* two-component system is adjacent to the major nitrate reductase encoded by the *narGHJI*-*1* operon, a prominent hypothesis is that this system is required for transcriptional regulation of the nitrate reductase, similar to the nitrate regulatory network of *P. aeruginosa* (Schobert and Jahn, 2010). Interestingly, while there is another predicted dissimilatory nitrate reductase encoded on chromosome II by the *narGHJI*-*2* operon, transposon mutant insertions within these loci do not function like *narGHJI*-*1* under the conditions tested. Moreover, we did not find evidence for homologs of the *narX/narL* two-component signaling system for nitrate sensing on chromosome II in our bioinformatics analyses.

**FIGURE 7:**
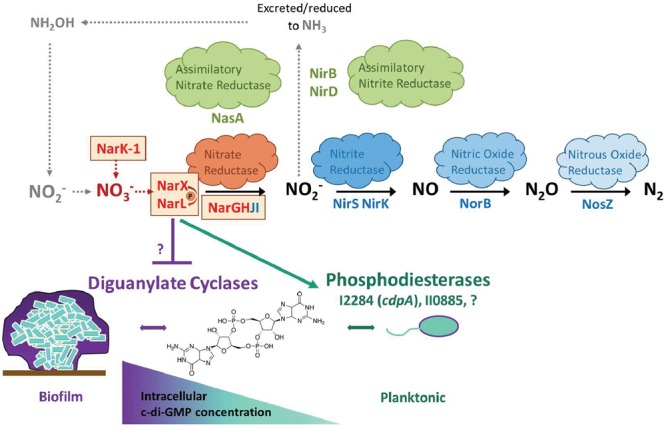
Working model for nitrate sensing and metabolism in relation to biofilm dynamics in *B. pseudomallei*. This model serves to illustrate nitrate metabolism in the context of biofilm dynamics for *B. pseudomallei*. The process of denitrification is carried out by four individual enzyme complexes: nitrate reductase, nitrite reductase, nitric oxide reductase, and nitrous oxide reductase. Additionally, *B. pseudomallei* encodes assimilatory nitrate and assimilatory nitrite reductases. The process of nitrification, depicted by gray arrows, is not intrinsic to *B. pseudomallei*; however, it is a relevant source of nitrate in the environment, produced by nitrifying bacteria. The five genes identified in this study are highlighted in bold red with surrounding boxes, in proximity to the major nitrate reductase (also highlighted in red). The biofilm life-cycle is illustrated as a state of constant flux mediated by intracellular c-di-GMP concentration. A proposed mechanism by which the NarX/NarL two-component nitrate-sensing system modulates c-di-GMP is illustrated, although the connection to diguanylate cyclases and additional phosphodiesterases remains to be determined.

*Burkholderia pseudomallei* is a facultative anaerobe that is found in soils approximately 30 cm deep (Palasatien et al., 2008), a depth which becomes relatively hypoxic in rice paddy soils (Andreae et al., 2014), and nitrate becomes increasingly more important as a terminal electron acceptor for anaerobic respiration. Similarly, biofilms are intrinsically hypoxic microenvironments as oxygen and nutrient diffusion are physically limited, leading to increased dependence on nitrate anions as an alternative terminal electron acceptor (Andreae et al., 2014). Based on our *in vitro* results, our working hypothesis is that excessive environmental nitrate triggers dispersal of hypoxic, nutrient-limited sessile cells residing in the biofilm community leading to increased abundance of planktonic bacteria. This working model is illustrated in the context of the denitrification pathway for *B. pseudomallei* 1026b and its potential connection to c-di-GMP metabolic regulatory cascades (**Figure 7**). It is important to mention that the information regarding c-di-GMP signaling and metabolism in *B. pseudomallei* is scarce, given the importance of this second messenger molecule in the regulation of biofilm dynamics (Plumley et al., 2017). Given the well-established connection between c-di-GMP and biofilm formation in many bacterial species, and the important transition from biofilm to planktonic cells in a variety of natural and host environments, it is vital to further identify and characterize the environmental cues that modulate c-di-GMP levels in the Tier 1 select agent *B. pseudomallei*.

The results presented here provide evidence that a primary mechanism for control of *B. pseudomallei* biofilm formation is through the regulation of the intracellular concentration of the bacterial second messenger, c-di-GMP, via nitrate sensing and metabolism. The role of c-di-GMP in the regulation of bacterial behaviors, which include biofilm formation, is known to have significant implications on the ability of pathogenic bacteria to cause disease (Tamayo et al., 2007). The model of c-di-GMP flux inside a cell generally dictates that more intracellular c-di-GMP molecules corresponds to a biofilm and sessile state, while a reduced intracellular concentration stimulates motility and favors a planktonic lifestyle; however, many of the extracellular cues that activate these signaling cascades remain uncharacterized (**Figure 7**) (Tamayo et al., 2007). Quantification of intracellular c-di-GMP levels in response to exogenous nitrate revealed that nitrate metabolism can affect the levels of this secondary messenger in *B. pseudomallei* 1026b. We hypothesize that the signaling transduction cascade initiated by nitrate sensing as controlled by the *narX/narL* two-component system activates not only dissimilatory nitrate reduction through the *narGHJI*-*1* complex but also regulates phosphodiesterase enzymes tasked with rapidly reducing intracellular concentrations of c-di-GMP. Our results indicate a significant reduction of c-di-GMP levels in static cultures grown in the presence of sodium nitrate, in addition to increased expression of *cdpA*, which encodes a known phosphodiesterase. These findings collectively indicate that the mechanism of nitrate sensing is linked to control of c-di-GMP levels in the cell.

Modulation of c-di-GMP levels is a primary mechanism bacteria use to respond to extracellular signals and cues as they relate to control of autoaggregation, biofilm formation, and motility during the transition from sessile to planktonic growth states. Therefore, such a mechanism is at the crux of controlling bacterial dissemination from environmental reservoirs to host-associated infections for sapronotic disease agents such as *B. pseudomallei.* CdpA, which has been characterized as a key phosphodiesterase in *B. pseudomallei* KHW (Lee et al., 2010) and at the genetic level in *B. pseudomallei* 1026b (Plumley et al., 2017), has also been implicated in the regulatory control of biofilm formation in *B. cepacia* J2315 (Ferreira et al., 2013). Our assessment of *cdpA* expression from *B. pseudomallei* 1026b revealed significant upregulation of this transcript in response to elevated nitrate availability, with a corresponding decrease of intracellular c-di-GMP levels. These data reveal an important connection between increased phosphodiesterase activity and reduction of this key second messenger molecule in response to the addition of nitrate. We expect global c-di-GMP metabolic cascades to be simultaneously activated by an exogenous nitrate signal, and given that we have previously identified 23 gene loci predicted to encode c-di-GMP metabolic or binding enzymes, there is undoubtedly more complex regulation in addition to *cdpA* activity (Plumley et al., 2017). Future studies will address nitrate’s global transcriptional regulation of c-di-GMP metabolism in addition to exopolysaccharide, capsule, pili, flagellar, quorum sensing, and antibiotic efflux machinery, among other mechanisms pertinent to the biofilm-to-planktonic transition.

The complexity of the regulatory network for denitrifying growth in hypoxic conditions has been extensively examined across several bacterial species (Campbell, 1977), implicating several transcriptional regulators of nitrate metabolism such as the *narX/narL* system in addition to quorum sensing and nutrient availability. Our study implicates nitrate metabolism in biofilm growth dynamics in *B. pseudomallei*, and identifies specific gene loci that may be targeted for regulation by a combination of systems. Furthermore, we provide evidence that the second messenger, c-di-GMP, is involved in biofilm inhibition in response to exogenous addition of nitrate. Future analyses will assess the regulation of the enzymes described in this study and investigate the effects of denitrification on c-di-GMP metabolism as it relates to the pathogenesis of *B. pseudomallei*.

## Author Contributions

MM and BB designed this study. MM and BP performed the experiments. MM analyzed the data and MM, BP, and BB wrote the paper. BB supervised data collection, analysis, and presentation of these results.

## Conflict of Interest Statement

The authors declare that the research was conducted in the absence of any commercial or financial relationships that could be construed as a potential conflict of interest.

## Footnotes

1 http://burkholderia.com

2 http://geneious.com

3 http://python.org
