# Supplementary material for: Nitrate Sensing and Metabolism Inhibit Biofilm Formation in the Opportunistic Pathogen Burkholderia pseudomallei by Reducing the Intracellular Concentration of c-di-GMP
Source: Front Microbiol. 2017 Jul 25;8:1353. doi: 10.3389/fmicb.2017.01353 (PMC5524735; doi:10.3389/fmicb.2017.01353)
Supplement: Supplementary file 1 [file Data_Sheet_1.DOCX]

Supplementary Material

Nitrate sensing and metabolism inhibit biofilm formation in the opportunistic pathogen *Burkholderia pseudomallei* by reducing the intracellular concentration of c-di-GMP

Mihnea R. Mangalea, Brooke A. Plumley, Bradley R. Borlee^*^

*** Correspondence:** Dr. Brad Borlee: Brad.Borlee@colostate.edu

# Supplementary Data

Supplementary Material should be uploaded separately on submission. Please include any supplementary data, figures and/or tables.

Supplementary material is not typeset so please ensure that all information is clearly presented, the appropriate caption is included in the file and not in the manuscript, and that the style conforms to the rest of the article.

# Supplementary Figures and Tables

## Supplementary Figures

##
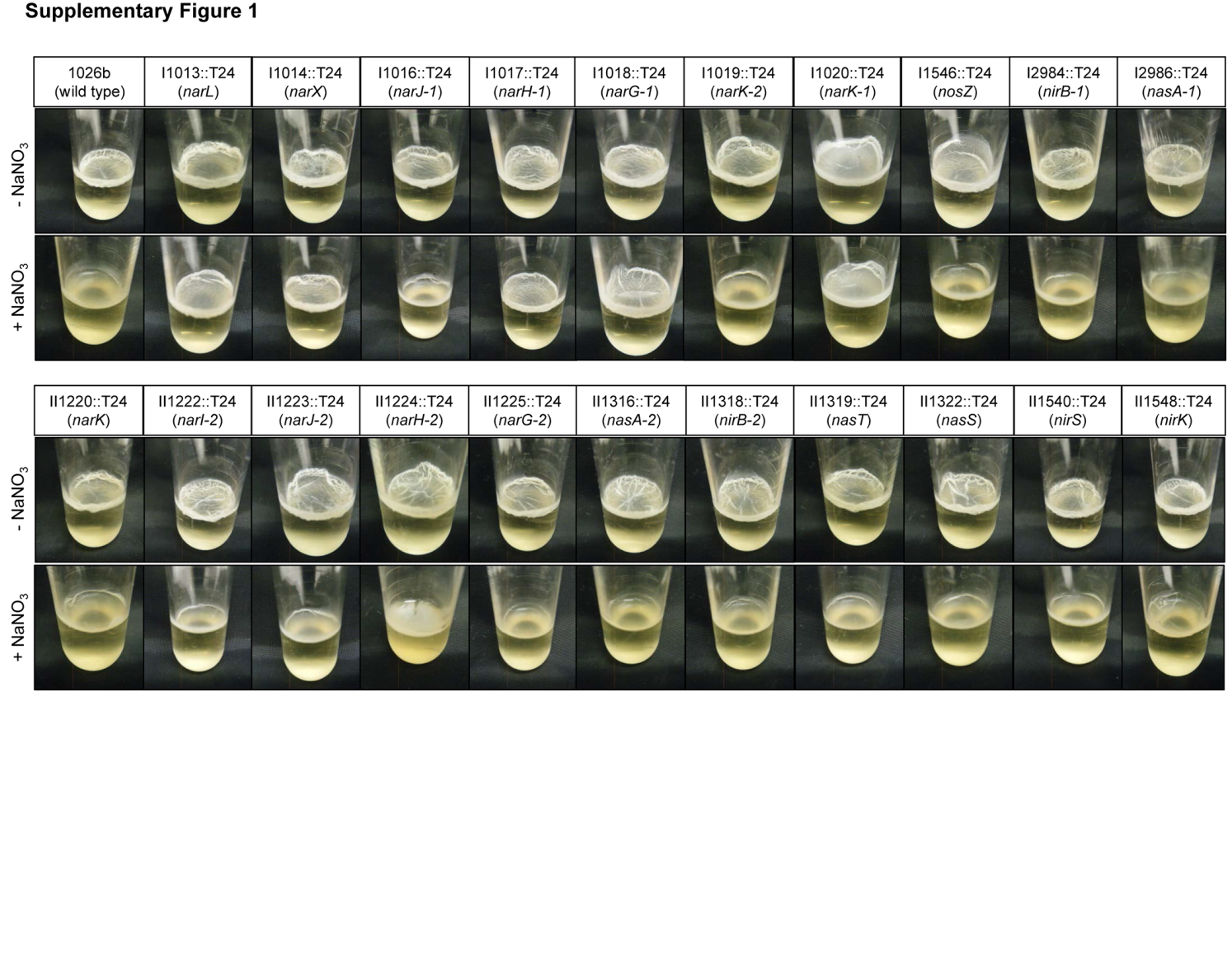


**Supplementary Figure 1. Assessment of pellicle biofilm formation for all 21 transposon insertional mutants used in this study.** Pellicle biofilms were grown statically in 3 mL LB medium with or without 10 mM NaNO_3_ supplemented and photographed at 14 days.


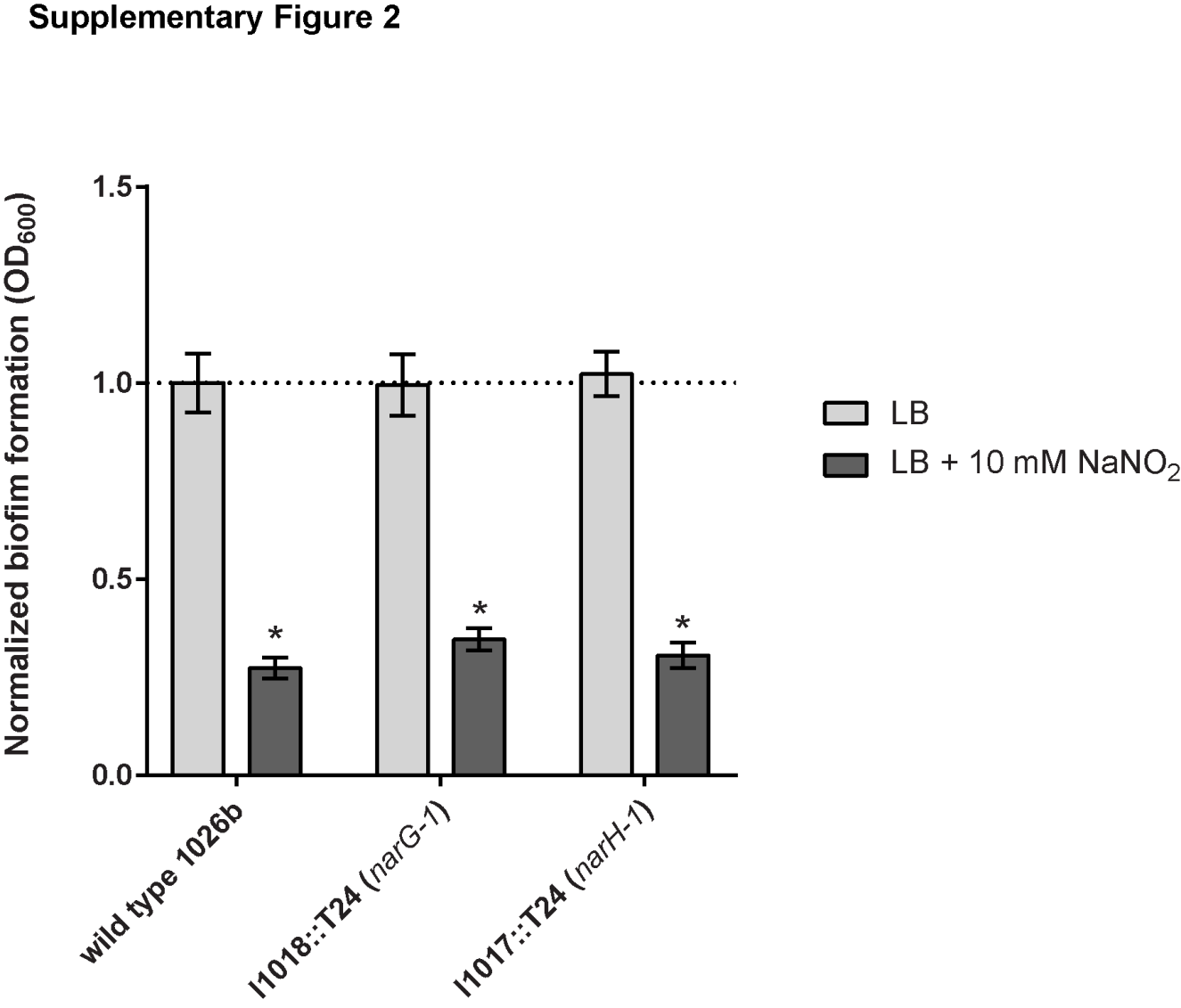


**Supplementary Figure 2. Biofilm inhibition by sodium nitrite does not require *narG-1* or *narH-1*.** Asterisks indicate a significant difference (p<0.0001) calculated with an unpaired Student’s t-test.

**
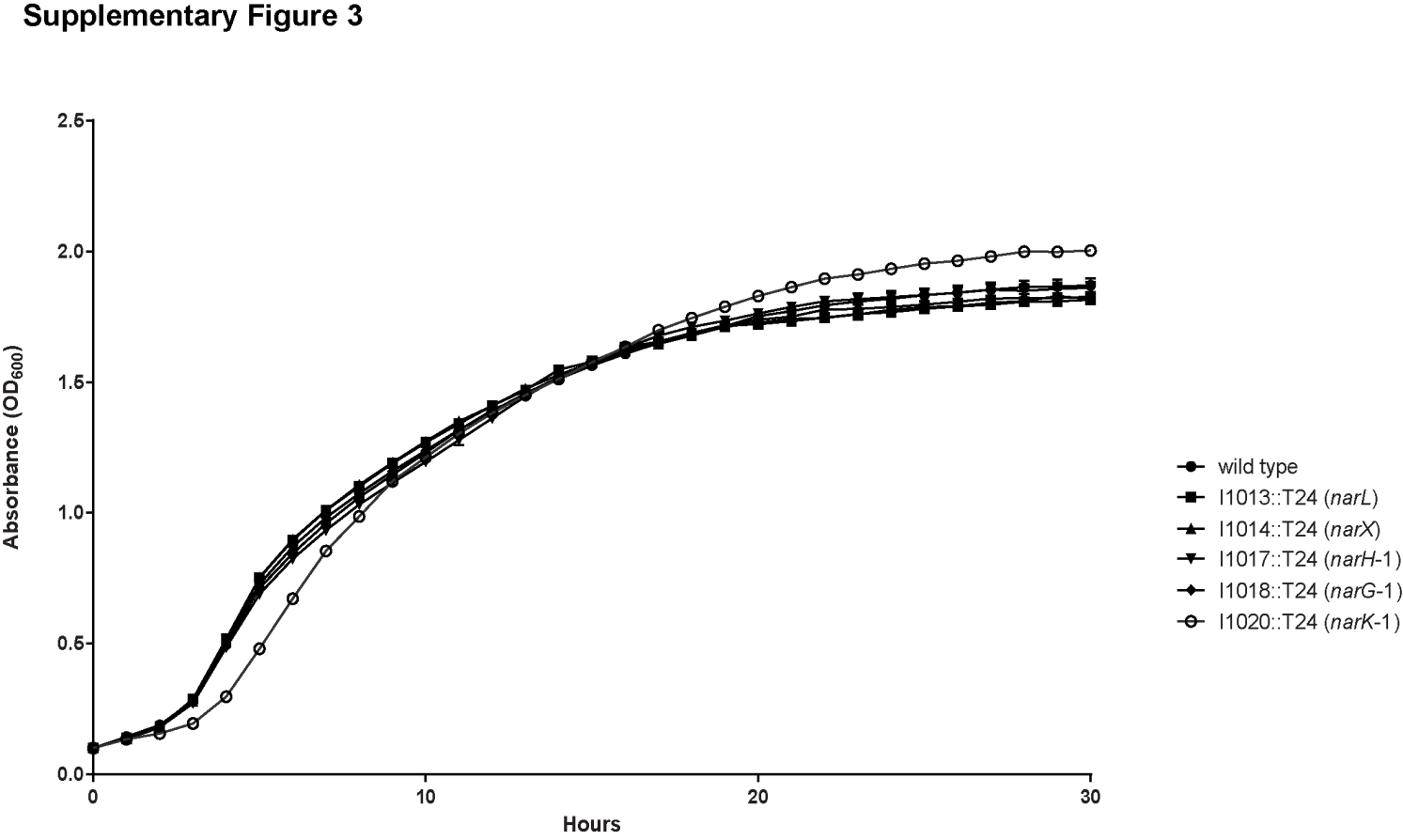
**

**Supplementary Figure 3. Growth curves for transposon insertion mutants in denitrification genes that no longer respond to nitrate mediated biofilm inhibition.** Growth curves were generated for cultures grown with shaking at 37°C and absorbance (OD_600_) readings were taken hourly for 30 continuous hours.

**
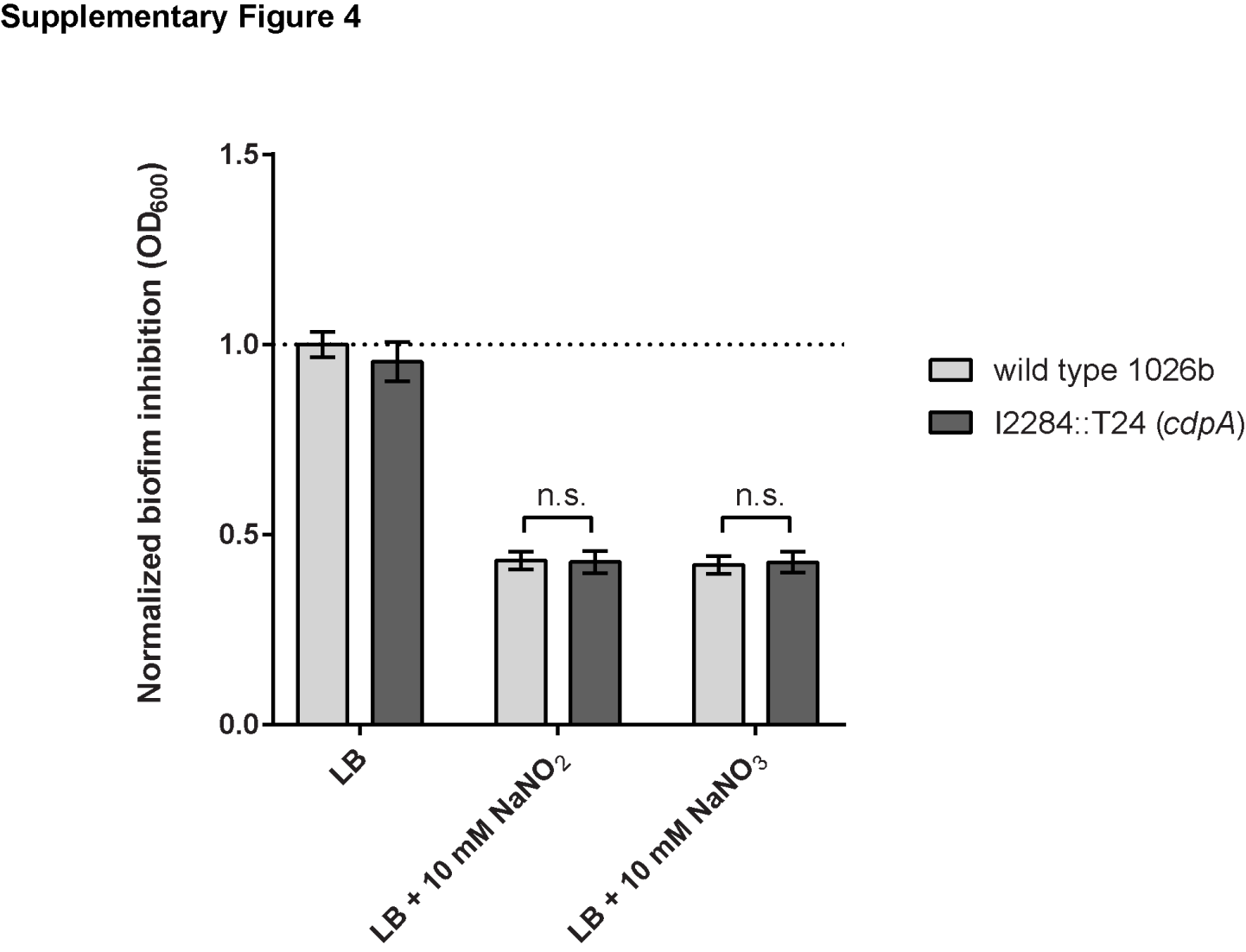
**

**Supplementary Figure 4. Biofilm inhibition by sodium nitrate or sodium nitrite does not require *cdpA*.**

## Supplementary Tables

**Supplemental Table 1. Predicted nitrogen metabolism genes in *Burkholderia* spp. in relation to *P. aeruginosa* PAO1.** Gene annotations and predicted functions were assembled using the Burkholderia Genome Database in conjunction with the Pseudomonas Genome Database. Sequence homologies were compared between *Burkholderia spp*. and *P. aeruginosa* PAO1. Locus identification for all genomes was determined using the NCBI GenBank database. Percent identities were calculated using the open-source MUSCLE alignment program from EMBL-EBI.


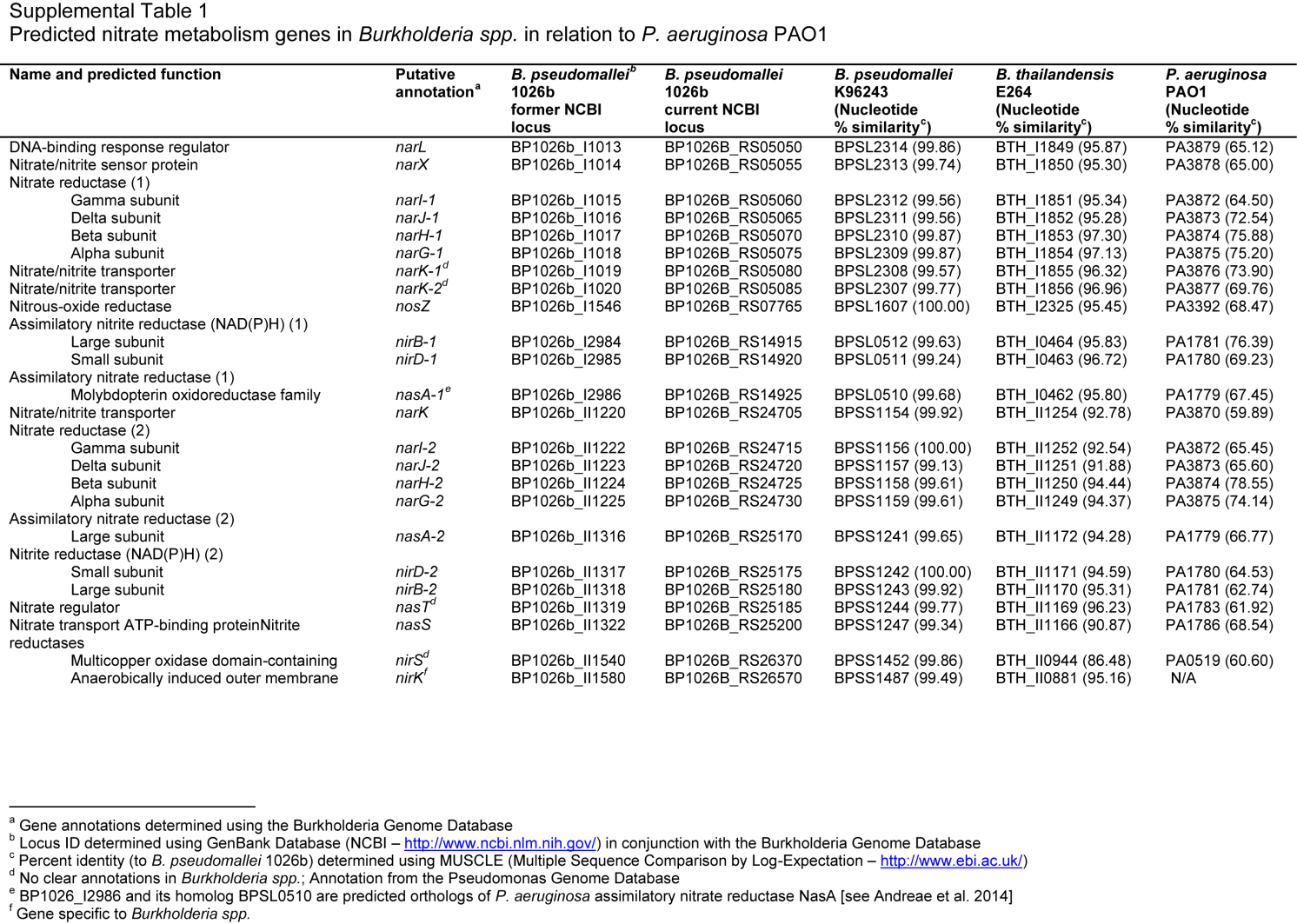


S**upplemental Table 2. Percent nucleotide identity of homologous nitrate metabolism genes between chromosomes I and II from *B. pseudomallei* 1026b.**

**
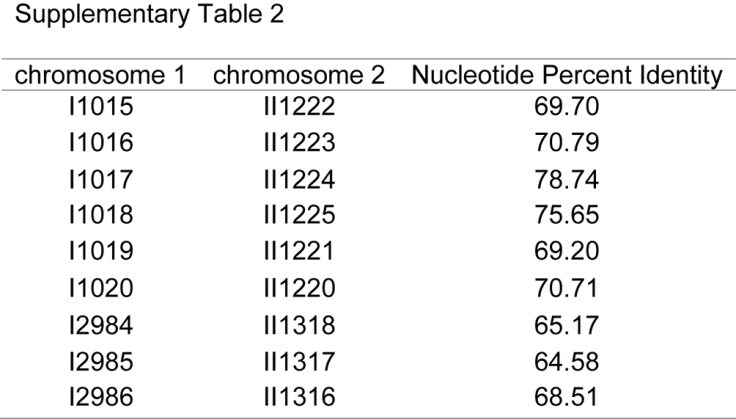
**
